# Totally Endoscopic Mitral Valve Repair for Barlow’s Disease in a Patient With Severe Pulmonary Dysfunction Following COVID-19 Pneumonia: A Case Report

**DOI:** 10.7759/cureus.90974

**Published:** 2025-08-25

**Authors:** Hitoki Hashiguchi, Naomi Yasuda, Akihito Ohkawa, Kyousuke Miki

**Affiliations:** 1 Cardiovascular Surgery, Hokkaido Prefectural Kitami Hospital, Kitami, JPN; 2 Cardiovascular Surgery, Sapporo Medical University, Sapporo, JPN

**Keywords:** barlow’s disease, covid-19, minimally invasive cardiac surgery, mitral valve repair, thoracoscopy

## Abstract

We report a case of totally endoscopic mitral valve repair for severe mitral regurgitation (MR) due to Barlow’s disease in a 55-year-old man with severe pulmonary dysfunction following coronavirus disease 2019 (COVID-19) pneumonia. He had developed severe COVID-19 one month earlier, requiring veno-venous extracorporeal membrane oxygenation (V-V ECMO). Although successfully weaned from ECMO, the patient continued to experience persistent fever and was referred to our department for suspected acute MR. Transthoracic echocardiography revealed severe MR with significant bileaflet prolapse.

Given the compromised pulmonary status and prior mini-tracheostomy, we opted for totally endoscopic mitral valve repair via a three-port approach using a 3D endoscope. Intraoperative findings confirmed no signs of infection but revealed characteristic thickening and billowing of both mitral leaflets, consistent with Barlow’s disease. Annuloplasty was performed using a 34-mm Physio Flex ring (Edwards Lifesciences, Irvine, CA, USA). The patient was extubated six hours after surgery without complications, and postoperative echocardiography confirmed complete resolution of MR. He was transferred back to the referring hospital on postoperative day nine for continued rehabilitation and steroid therapy.

This case suggests that totally endoscopic mitral valve repair may be a feasible and safe option even for patients with severe pulmonary dysfunction following COVID-19-related lung injury.

## Introduction

Coronavirus disease 2019 (COVID-19) can cause severe pneumonia and acute mitral regurgitation in rare cases [1], and may require mechanical ventilation or extracorporeal membrane oxygenation (ECMO), with sequelae that include long-term pulmonary dysfunction such as impaired diffusion capacity, interstitial fibrosis, and hypoxemia [2]. These complications present unique perioperative challenges, especially for cardiac surgery requiring single-lung ventilation. Minimally invasive cardiac surgery (MICS) through a thoracoscopic approach has been reported to reduce surgical trauma, facilitate early mobilization, and shorten pulmonary recovery compared with median sternotomy [3].

Barlow’s disease is a degenerative form of mitral valve prolapse characterized by bileaflet prolapse, excess leaflet tissue, and annular dilation. It remains technically demanding for mitral valve repair, though several reports demonstrate that annuloplasty alone may provide durable results in selected cases [4-6]. For general clinicians unfamiliar with this entity, it is important to note that Barlow’s disease can remain clinically silent until stressors such as infection or myocardial injury trigger acute decompensation. In patients with concomitant COVID-19-associated interstitial lung disease (ILD), careful surgical indication and risk-benefit analysis are essential [2]. This case highlights the feasibility of totally endoscopic mitral valve repair in such a high-risk context, with particular emphasis on surgical indication, perioperative management of impaired lung function, and the rationale for choosing a thoracoscopic approach over sternotomy.

## Case presentation

A 55-year-old man with no prior history of cardiac disease or dyspnea developed severe COVID-19 pneumonia in October 2021, requiring intubation and veno-venous (V-V) ECMO support for seven days. He recovered from ECMO but continued to experience persistent low-grade fever. At follow-up, a new systolic murmur was identified, which had not been documented on previous medical check-ups. His dyspnea worsened progressively after COVID-19 pneumonia, despite resolution of the acute infection.

**Figure 1:**
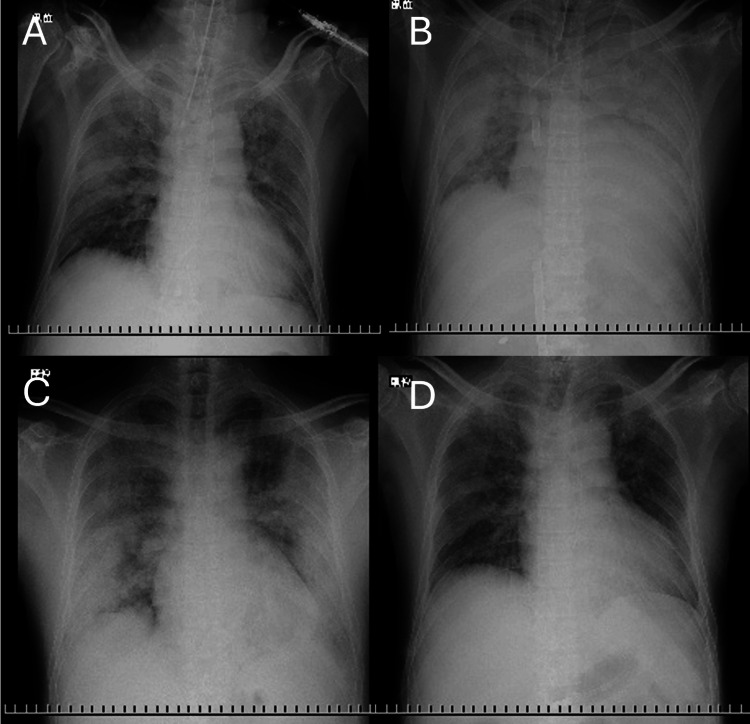
Serial chest X-rays from admission to transfer. (A) Immediately after admission during mechanical ventilation. (B) At the time of V-V ECMO initiation. (C) After weaning from V-V ECMO. (D) Upon transfer to our department, showing residual diffuse bilateral opacities.

**Figure 2:**
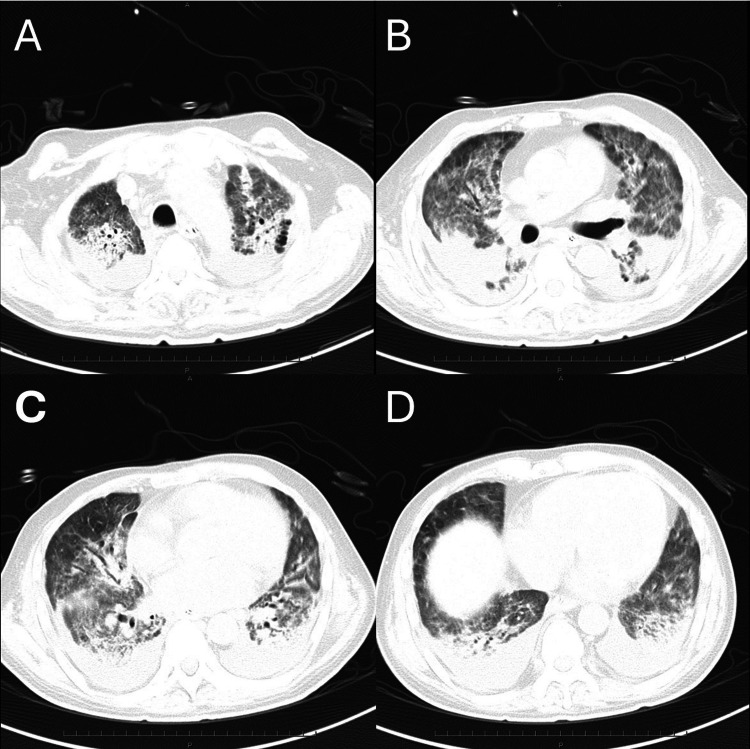
Preoperative chest computed tomography (CT) scans. Preoperative chest CT images from the apex (A) to the lung base (D) demonstrate extensive lung infiltration following severe COVID-19 pneumonia.

On admission to our department, the patient was afebrile but tachypneic, with SpO₂ 92% on room air. Auscultation revealed a grade IV/VI mid-systolic murmur at the apex. Chest X-ray (Figure 1) demonstrated the chronological course: (A) immediately after admission during mechanical ventilation, (B) at the time of V-V ECMO initiation, (C) after weaning from V-V ECMO, and (D) upon transfer to our department, showing diffuse bilateral opacities, and chest CT (Figure 2) showed bilateral interstitial infiltrates consistent with post-COVID ILD.

Because the patient was under respiratory compromise and infective endocarditis (IE) was initially suspected, only limited transthoracic echocardiogram (TTE) could be performed at the bedside. Therefore, detailed quantitative assessment was not feasible. Importantly, IE could not be definitively excluded until intraoperative inspection.

**Figure 3:**
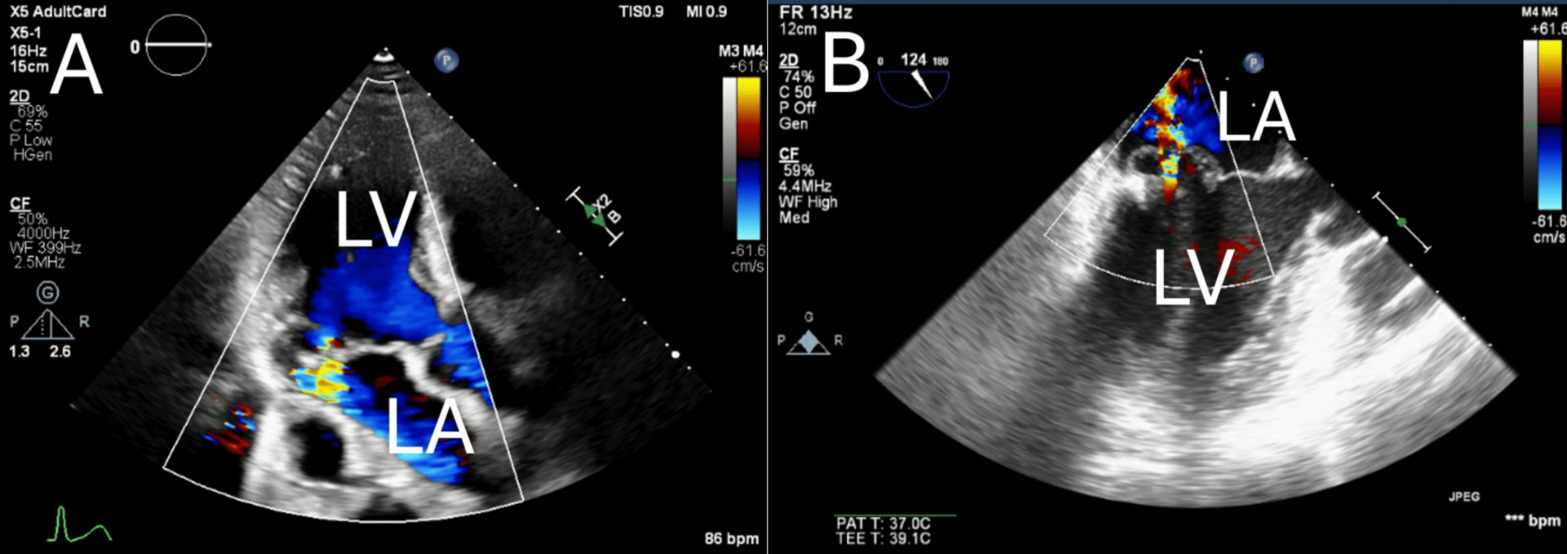
Preoperative echocardiographic findings. (A) Transthoracic echocardiography (TTE) before surgery shows an eccentric jet suggesting anterior leaflet prolapse. (B) Intraoperative transesophageal echocardiography (TEE) before repair reveals prolapse-like motion of both leaflets, consistent with Barlow’s disease.

TTE (Figure 3A) demonstrated severe MR with bileaflet prolapse, and intraoperative transesophageal echocardiography (TEE) (Figure 3B) confirmed these findings. Laboratory data included leukocytosis, anemia, thrombocytosis, elevated C-reactive protein (CRP), and lactate dehydrogenase (LDH) elevation. At the time of transfer to our department, the patient was managed with high-flow nasal therapy (FiO₂ 40%, 40 L/min). The PaO₂/FiO₂ ratio was reduced, indicating impaired oxygenation and low pulmonary function. KL-6 and SP-D were measured as part of the preoperative evaluation; there was no marked increase in KL-6, and only a mild elevation in SP-D was noted. These findings are summarized in Table 1.

**Table 1:** Key Laboratory Data at Admission CRP: C-reactive protein; ALT: alanine aminotransferase; AST: aspartate aminotransferase; eGFR: estimated glomerular filtration rate; NT-proBNP: N-terminal pro-B-type natriuretic peptide; LDH: lactate dehydrogenase,

Test Item	Reference Range	Unit	Result
WBC	3.30–8.60	×10³/µL	10.48
RBC	4.35–5.55	×10⁶/µL	3.61
Hemoglobin	13.7–16.8	g/dL	10.7
Hematocrit	40.7–50.1	%	34.4
Neutrophils	39.8–70.5	%	83.8
Lymphocytes	23.1–49.9	%	5.8
Platelets	158–348	×10³/µL	393
CRP	0.00–0.14	mg/dL	0.3
AST	13–30	U/L	28
ALT	10–42	U/L	82
eGFR	60.0–999	mL/min/1.73 m²	118.1
NT-proBNP	0–54.9	pg/mL	169.7
LDH	124–222	U/L	280
pCO₂	35–45	mmHg	37.9
pO₂	80–100	mmHg	72.5
HCO₃⁻	22–26	mmol/L	31.5
SO₂	95–100	%	95
pH	7.35–7.45		7.529
KL-6	<500	U/mL	303
SP-D	<110	ng/mL	251

**Figure 4:**
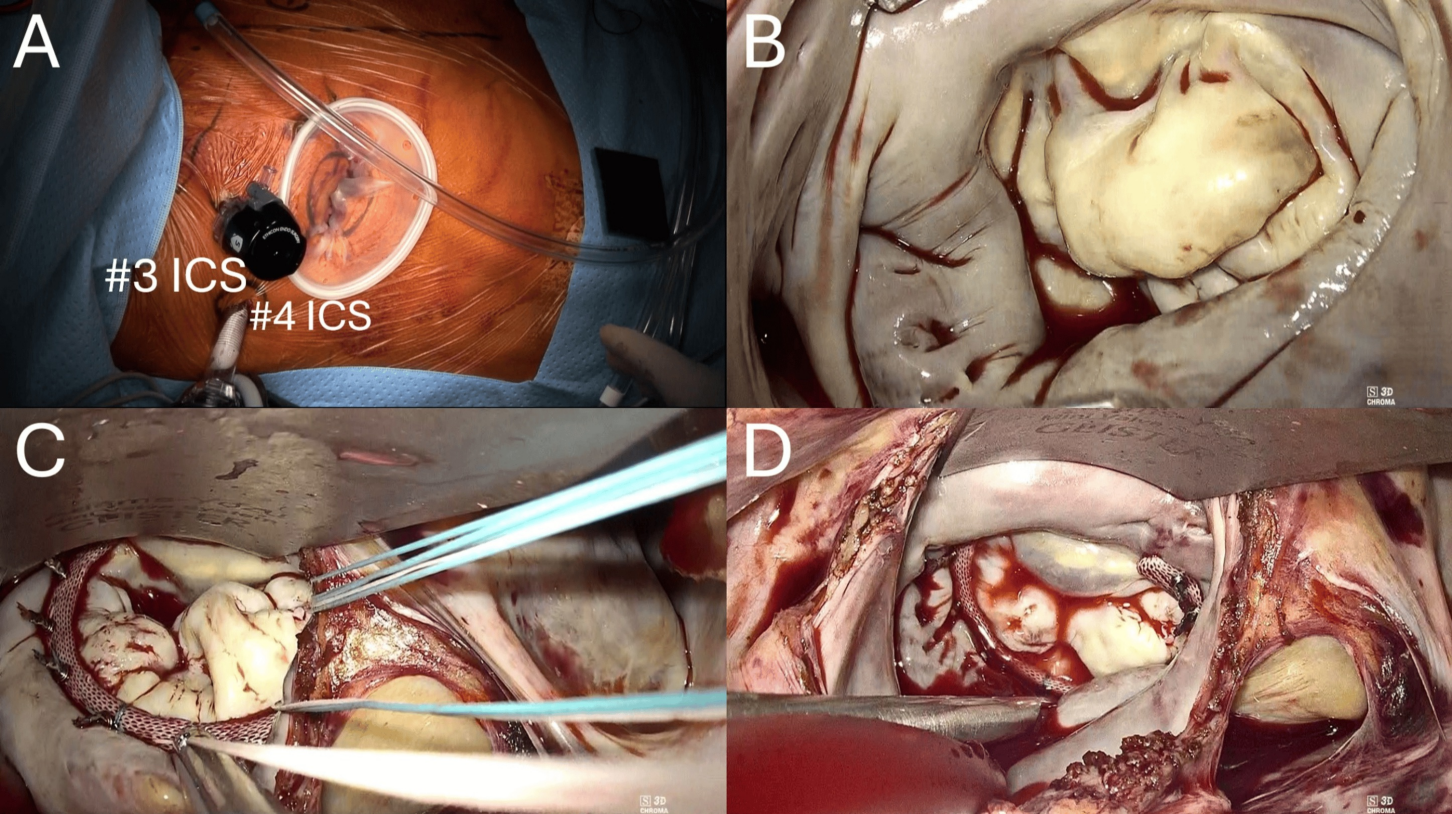
Intraoperative findings. (A) Port placement for totally endoscopic mitral valve repair. (B) Intraoperative view of the mitral valve via the left atrium. The entire leaflet structure shows severe thickening, characteristic of Barlow’s disease. (C) Annuloplasty sutures being secured using CorKnot. (D) Water test after annuloplasty confirms the absence of regurgitation.

**Figure 5:**
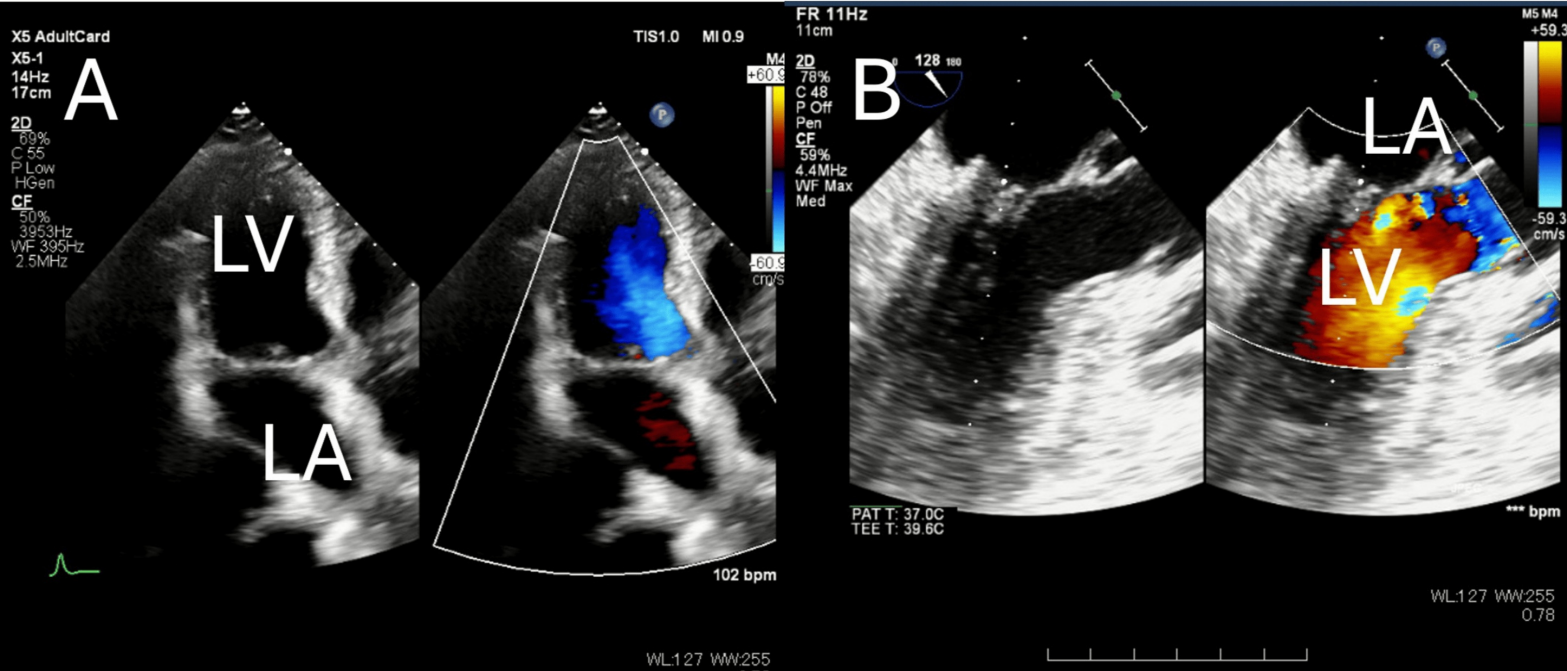
Postoperative echocardiographic evaluation. (A) Transthoracic echocardiography (TTE) after surgery demonstrates no residual mitral regurgitation. (B) Intraoperative transesophageal echocardiography (TEE) following repair also confirms the complete elimination of regurgitation.

Following these findings, surgical intervention was pursued. A totally endoscopic mitral valve repair was performed via a right mini-thoracotomy three-port approach, with cardiopulmonary bypass established through femoral cannulation. Intraoperative findings (Figure 4) showed thickened, redundant mitral leaflets typical of Barlow’s disease, without evidence of infective endocarditis. Annuloplasty with a 34-mm Physio Flex ring (Edwards Lifesciences, Irvine, CA, USA) was performed, and intraoperative water test and TEE (Figure 5) confirmed elimination of MR. The operative time was 354 minutes, cardiopulmonary bypass and cross-clamp times were 203 and 117 minutes, respectively, and estimated blood loss was 150 mL.

The patient was extubated six hours postoperatively and had an uneventful recovery. Temporary pacing wires were removed on day one and chest drains on day three. Oral intake was resumed on day three. The tracheostomy tube was not reinserted into the patient. He was transferred back to the referring hospital on postoperative day nine for continued corticosteroid therapy and pulmonary rehabilitation after extubation.

## Discussion

Pulmonary sequelae following COVID-19, particularly in patients requiring ECMO, often present as interstitial changes with impaired diffusion capacity rather than simple restrictive physiology [2]. This distinction is important because such patients may poorly tolerate one-lung ventilation. Prior comparative studies indicate that minimally invasive thoracoscopic surgery may allow faster recovery of pulmonary function than sternotomy, supporting our choice of approach in this high-risk patient [3].

In the present case, the patient demonstrated bilateral interstitial changes and mildly elevated SP-D, suggesting residual inflammatory activity. Despite reduced oxygenation reflected by a low PaO₂/FiO₂ ratio, careful perioperative management - including intermittent two-lung ventilation and early extubation - enabled successful completion of totally endoscopic repair without postoperative pulmonary complications.

The etiology of acute MR was bileaflet prolapse associated with Barlow’s disease. However, the clinical course suggests that COVID-19 may have acted as a precipitating factor. Several reports have described acute MR in COVID-19 patients due to myocarditis or papillary muscle dysfunction [1]. We therefore consider that COVID-related myocardial involvement and systemic inflammation may have accelerated the deterioration of previously silent Barlow’s disease.

Another important aspect is surgical indication and perioperative management. Patients with interstitial lung disease after COVID-19 are considered at particularly high risk for acute exacerbation after surgery. Previous Japanese studies have proposed risk scoring systems for acute exacerbation of ILD, incorporating factors such as preoperative history of acute exacerbation, CT findings of usual interstitial pneumonia (UIP) pattern, steroid use, high KL-6, and reduced vital capacity [7]. These risk stratification tools are useful in determining candidacy and tailoring perioperative strategy. In our patient, favorable preoperative performance status and preserved gas exchange under high-flow oxygen supported the surgical indication. Intraoperative strategies included intermittent two-lung ventilation and minimizing surgical trauma through a totally endoscopic approach, both of which facilitated early extubation and an uneventful recovery.

Repair of MR due to Barlow’s disease is technically demanding because of bileaflet prolapse and excess leaflet tissue. Nonetheless, several series have reported that annuloplasty alone can stabilize the posterior annulus and yield durable repair in carefully selected patients [4,5]. In our case, the absence of chordal rupture or leaflet perforation supported the decision to perform annuloplasty alone, and postoperative echocardiography confirmed the adequacy of this strategy.

This case, therefore, illustrates that totally endoscopic mitral valve repair can be safely performed in selected high-risk patients with COVID-19-related pulmonary dysfunction, provided that comprehensive risk assessment and meticulous perioperative management are undertaken.

## Conclusions

Totally endoscopic mitral valve repair using a 3D thoracoscopic approach was successfully performed in a patient with Barlow’s disease complicated by COVID-19-related pulmonary dysfunction. This approach minimized surgical trauma, enabled early extubation, and avoided reintubation, suggesting it may be a viable option even in high-risk patients. Further studies are needed to evaluate long-term durability and applicability to broader patient populations.
